# Economic burden of lives lost due to COVID-19 in California State

**DOI:** 10.2217/cer-2021-0245

**Published:** 2022-03-10

**Authors:** Michelle Zheng, Briana Lui, Axell-Giovanni A Komlan, Christina R Bonaparte, Robert S White, Marguerite M Hoyler

**Affiliations:** ^1^Cornell University, College of Human Ecology, Martha Van Rensselaer Hall, Ithaca, NY 14850, USA; ^2^Department of Anesthesiology, New York-Presbyterian/Weill Cornell Medical Center, 525 East 68th Street, Box 124, New York, NY 10065, USA; ^3^University of Notre Dame, College of Science, Candidate for Bachelor of Science in Science Preprofessional Studies, Notre Dame, IN 46556, USA; ^4^Brown University, Candidate for Bachelor of Arts in Public Health with Honors, Providence, RI 02912, USA

**Keywords:** California, COVID-19, health economics, value of statistical life, years of potential life lost

## Abstract

**Aim:** To examine the economic impact of lives lost due to the coronavirus pandemic across California and Los Angeles (LA) County. **Patients & methods:** Years of potential life lost (YPLL) and the value of statistical life (VSL) were calculated using mortality data from the California Department of Public Health, the LA County Department of Public Health and the Social Security Administration websites. **Results:** In California and LA County, the average YPLL per person were 14.3 and 14.7 and the VSLs were approximately US$219.9 billion and $82.7 billion, respectively. YPLL and VSL were greatest for Latinos aged 50–64. **Conclusion:** The economic burden of lives lost due to the coronavirus across California and LA County is substantial. Latinos aged 50–64 were most affected.

The COVID-19 pandemic has created an unprecedented, adverse and indelible impact on countless individuals, family units and communities worldwide. In the USA, as of August 2021, there were a reported 35,392,284 infections and 612,958 mortalities, and 9.6 million American workers lost their jobs [1]. Racial minorities and socioeconomically disadvantaged groups have borne the brunt of the pandemic's most adverse economic outcomes. According to the National Low Income Housing Coalition, as of October 2020, 1 in 4 adults have struggled to pay their bills since the start of the pandemic, including 43% of Black adults, 37% of Hispanic adults and 46% of lower-income adults [2]. Black and Hispanic workers also make up greater shares of the front-line workforce, which puts them and their families at risk for contracting COVID-19 [3].

Excess mortality due to COVID-19 has had a significant economic impact on the United States, as measured by metrics such as years of potential life lost (YPLL) and the value of statistical life (VSL) [4]. YPLL is used to estimate premature mortality and the number of years lost from a predefined age, such as life expectancy, with a higher YPLL suggestive of premature mortality [5]. VSL is used by economists as a standard measure to evaluate the financial costs and cost–effectiveness of potentially life-saving and life-prolonging policies and interventions [6].

California has the largest state economy [7] and the largest, most diverse population in the USA, with over 39 million residents [8]. As of August 2021, California reported the highest number of COVID-19 cases and deaths in the United States, with nearly 3.9 million cases, or 11.0% of the national total, and approximately 10.5% of total deaths (63,891 deaths) [9]. Los Angeles (LA) County carried the majority of these cases (1.4 million, or 32.5%) and deaths (23,380, or 36.6%) [10].

Examining the economic burden of COVID-19 mortality in California provides an important view of the cost of the pandemic in a major US economy. The aim of this paper is to estimate the YPLL and the VSL to better understand the economic cost of COVID-19 mortality in the state of California and in LA County, as well as across racial and ethnic minority groups in those regions.

## Methods

### Data acquisition

Cumulative state- and county-level coronavirus mortality data were retrieved from the California Department of Public Health and the LA County Department of Public Health websites on 28 July 2021 and 30 July 2021, respectively [11,10]. Extracted data included all known COVID-19 death counts by age range dating back to 1 March 2020 for the state of California and LA County, excluding Long Beach and Pasadena. Long Beach and Pasadena each have their own respective health departments and are not served under the LA County Department of Public Health [10]. Mortality data by age range were further stratified by race and ethnicity for the state of California [12].

Period life expectancy was extracted from the most recent (2017) actuarial life tables from the Social Security Administration website [13]. Period life expectancy is defined as the average number of years of life remaining if a group of persons at that age were to experience the mortality rates for 2017 over the course of their remaining life [13]. Acquired data included the average number of years of life remaining for males and females from birth to 119 years of age. The actuarial life tables reflect values for the Social Security area population, which includes residents of the 50 states and the District of Columbia (adjusted for net census undercount); civilian residents of Puerto Rico, the Virgin Islands, Guam, American Samoa and the Northern Mariana Islands; federal civilian employees and persons in the US Armed Forces abroad and their dependents; non-citizens living abroad who are insured for Social Security benefits; and all other US citizens abroad [13]. All extracted data were publicly available and did not require approval from the institutional review board.

### Statistical analysis

The baseline age of death was calculated by using the median or higher of two midpoint values for each age range (e.g., 11 for the age range 5–17). The age ranges for California State were 0–4, 5–17, 18–34, 35–49, 50–59, 60–64, 65–69, 70–74, 75–79 and 80 and over. The age ranges for LA County, excluding Long Beach and Pasadena, were 0–4, 12–17, 18–29, 30–49, 50–64, 65–79 and 80 and over. For California State COVID death counts further stratified by race and ethnicity, the age ranges were 0–17, 18–34, 35–49, 50–64, 65–79 and 80 and over. For 80 and over, the age range was set to 80–100 with a midpoint of 90.

The primary outcomes analyzed by this study were the YPLL and the VSL due to COVID-19 in California State and LA County, excluding Long Beach and Pasadena. The secondary outcomes were YPLL and VSL further stratified by race and ethnicity for California State only. The number of COVID-19 deaths in each age range was multiplied by the average period life expectancy for males and females at the midpoint of each age range to estimate the YPLL. The VSL for each age range was calculated by multiplying the YPLL by the population average value of statistical life year (VSLY). The VSLY was determined to be US$240,676 based on previously published literature and a VSLY methodology that incorporated age range by decile and income quintile [14]. This VSL could be used by policymakers to help estimate the economic burden of premature deaths due to the coronavirus pandemic and evaluate the cost-benefit ratio of different risk-reduction interventions. The YPLL and VSL values of each age range were summated to determine a total YPLL and VSL value. Total YPLL was then divided by the total number of deaths (including any unknown or missing deaths) to determine the average YPLL per person. Upper and lower limit YPLL and VSL sensitivity analyses for California State and LA County were calculated by replacing the midpoint value of the average period life expectancy with the upper and lower end of each respective age range [4].

## Results

A total of 63,891 coronavirus deaths had been reported across the state of California as of 28 July 28 2021. These deaths resulted in an estimated 913,682 YPLL (range: 770,352–1,126,069) and approximately US$219.9 billion VSL (range: US$185.4–$271 billion) (Table 1 & Figure 3 & 4). The mean YPLL per person was 14.3 (range: 12.1–17.6). Deaths from the 35–49, 50–59 and 60–64 age ranges contributed to almost half of the total YPLL, at 14.6%, 20.4% and 13.8%, respectively. By race and ethnicity, Latinos accounted for over half of the total YPLL and VSL, at 56.5% and 64.9%, respectively, followed by Whites, Asians and African Americans (Table 4, Figures 5& 6).

**Table 1.** Total years of potential life lost and value of statistical life for California and Los Angeles County, excluding Long Beach and Pasadena.

Deaths	California			Los Angeles County (excluding Long Beach and Pasadena)		
	63,891			23,380		
	Total YPLL	Average YPLL (per person)	Total VSL (USD)	Total YPLL	Average YPLL (per person)	Total VSL (USD)
Base case	913,682	14.30	$219,901,208,700	343,186	14.68	$82,596,749,260
Lower bound	770,352	12.06	$185,405,180,200	249,297	10.66	$59,999,836,060
Upper bound	1,126,069	17.62	$271,017,717,700	465,948	19.93	$112,142,390,100

USD: US dollars; VSL: Value of statistical life; YPLL: Years of potential life lost.

**Figure 1.**
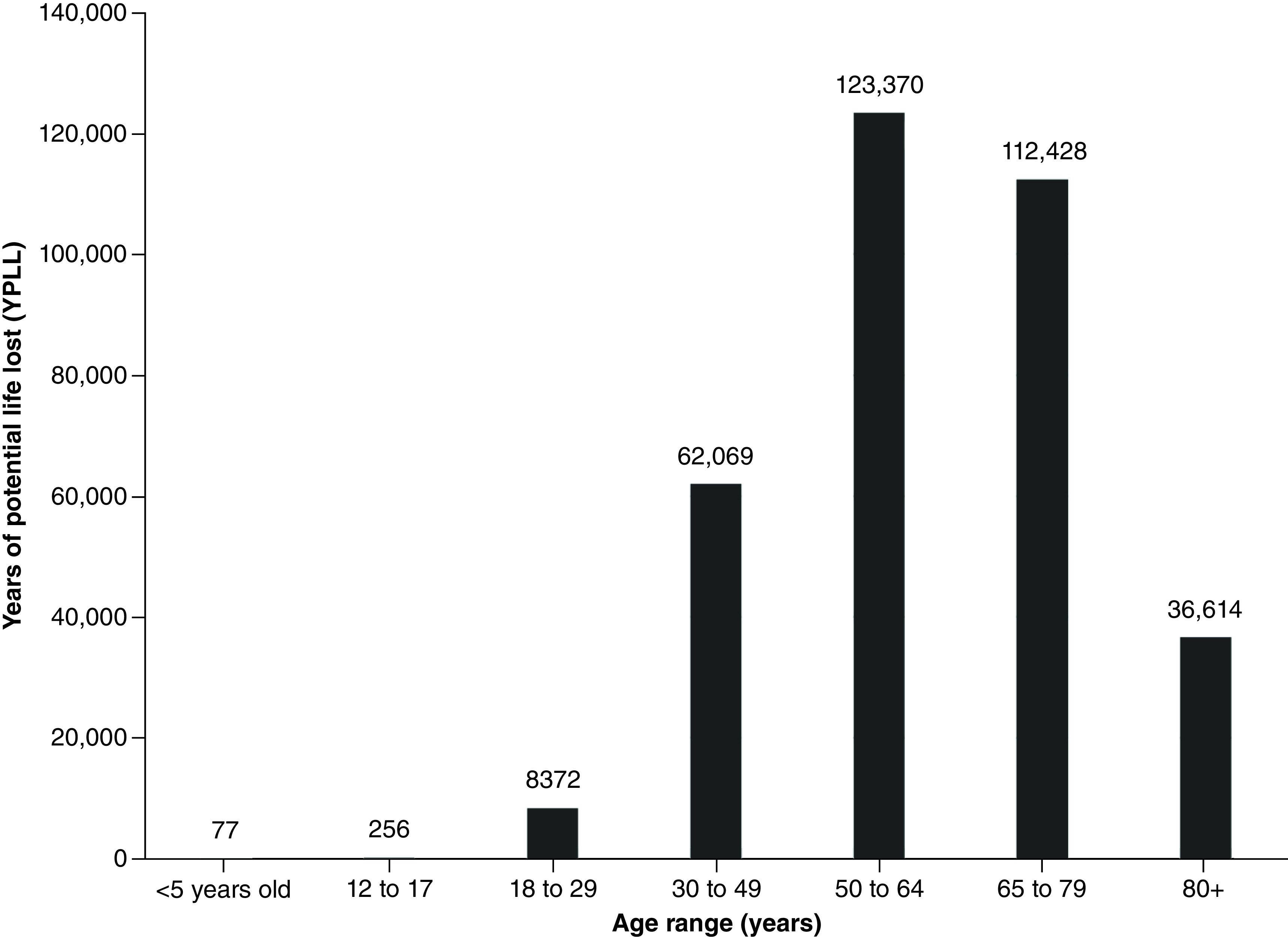
Years of potential life lost by age range for Los Angeles County, excluding Long Beach and Pasadena.

**Figure 2.**
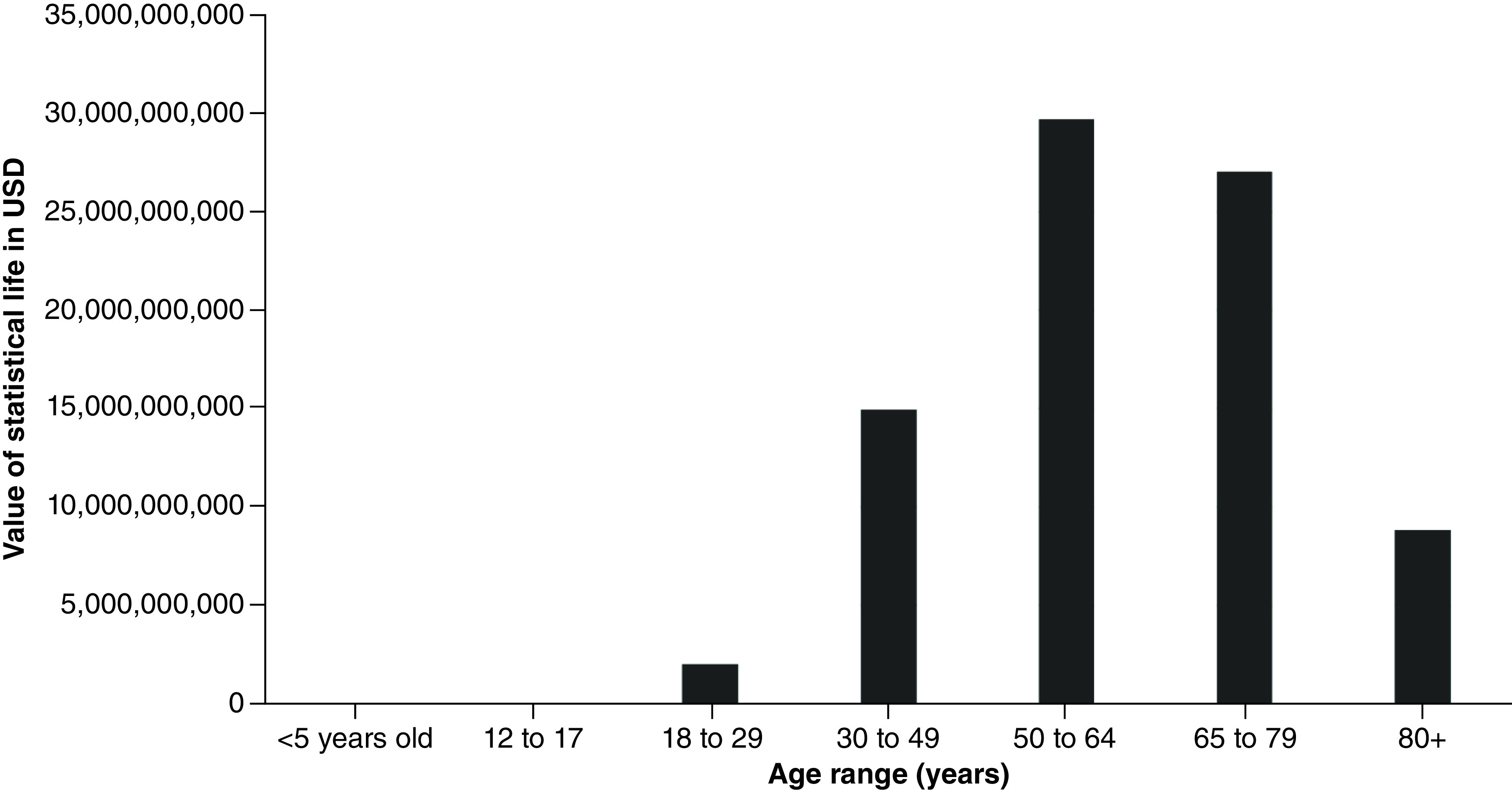
Value of statistical life by age range for Los Angeles County, excluding Long Beach and Pasadena. USD: US dollars.

In LA County, excluding Long Beach and Pasadena, coronavirus deaths totaled 23,380 as of 30 July 2021. These deaths contributed to an estimated 343,186 YPLL (range: 249,297–465,948) and approximately US$82.7 billion VSL (range: US$59.6–$112.1 billion). The average YPLL per person was 14.7 (range: 10.7–19.9) (Table 1). Fatalities from the 50–64 and 65–79 age ranges contributed to more than half of the total YPLL and VSL, at 36% and 32.7%, respectively (Figures 1 & 2).

## Discussion

This study estimated the VSL lost and the YPLL in California State and LA County, excluding Long Beach and Pasadena, from the beginning of the pandemic (1 March 2020) through July 2021. Across California, the monetary value of lives lost exceeded US$219.9 billion, with LA County accounting for roughly US$82.7 billion, as of 28 July 2021. Among all California counties, LA County was disproportionately impacted, with nearly five-times as many deaths as the next highest county [15]. These findings may reflect a combination of factors, including population density, poverty, race and ethnicity, homelessness and high household densities in LA County compared with other areas of the state [16]. Analysis by age range determined that older individuals accounted for most of the monetary cost of lives lost due to this pandemic, in spite of the fact that older populations tend to have fewer years of life remaining. These findings are consistent with prior literature demonstrating that older individuals over 50, and especially over 60, face elevated mortality risk due to COVID-19 [17].

Sub-analysis by race and ethnicity further indicated that certain racial and ethnic minority groups were more heavily impacted by the COVID-19 pandemic. Latinos comprise 39.4% of the population of California State but accounted for the greatest economic burden of lives lost (56.5% and 64.9% of YPLL and VSL respectively) [9]. They had the highest total YPLL and VSL, at 516,288 and $124.3 billion, respectively. Similarly, African Americans make up only 6.5% of California's population yet had a higher total YPLL (62,010) than Asians and Whites, who account for 15.5% and 36.5% of California's population, respectively [18]. These differences may be due to a combination of environmental and health factors. Individuals of color are more likely to have poor access to healthcare and underlying health conditions, such as asthma, diabetes and hypertension, which increase their risk of COVID-19 complications and fatality [19]. Compared with Whites and Asians, Latinos and African Americans are more likely to live in more crowded households with insufficient space for social distancing and self-isolation [20]. Furthermore, individuals of color are more likely to work in service and public-facing jobs that do not offer the ability to telework, thereby increasing their vulnerability and susceptibility to COVID-19 infection [19]. Latinos and African Americans are also more likely to live in poverty [21]. Prior literature has identified an association between low socioeconomic status, crowded households and an inability to telework and a disproportionate COVID-19 positivity and mortality rate among communities of color in California [22,23]. Specifically, prior literature has shown an increased rate of hospitalizations among individuals of color due to COVID-19, even after adjusting for comorbidities, highlighting the impact of social inequality during this pandemic [23].

With nearly 40 million residents, California has the largest population of any US state; it is also among the youngest and most diverse, with more Latino than White residents since 2014 [21]. California also accounts for 15% of the US economy [24]. In this context, the magnitude of the financial burden of COVID-19 mortality in California, as well its disproportionate impact on non-White communities, is particularly significant and concerning.

The results of the present study are consistent with similar disparities analyzed in New York, Ohio and Illinois. In New York and Ohio, older individuals bore the greatest economic burden of life lost, with individuals over the age of 60 experiencing the greatest impact [4,25]. In Chicago, Illinois, and in Cook County in general, Black and Hispanic residents experienced a disproportionate COVID-19 burden and premature loss of life [26,27]. In Cook County specifically, Latinos had a YPLL rate 4.8-times greater than Asians, the least affected group [26]. In Chicago, geospatial analysis identified COVID-19 mortality hot spots in neighborhoods of color and low socioeconomic status as well as higher rates of chronic comorbidities [27]. While the current data cannot be directly extrapolated to other states, the current findings are worrisome for similar trends across the USA.

The current study has several limitations. Most notably, the values included here were current as of July 2021; they do not reflect the full extent of the pandemic, as new fatality reports are confirmed and updated daily, particularly in the setting of new and more transmissible variants [28]. Furthermore, the COVID-19 pandemic has led to an excess of non-COVID deaths, related to factors such as delays in or avoidance of medical care, drug use and mental health crises [29,30]. As a result, the calculated YPLL and VSLs are surely an underestimation of the current economic losses and loss of life experienced across California and LA County due to COVID-19. Additionally, some data were incomplete at the time of analysis: 566 fatality reports listed an unknown or ‘missing’ age (Tables 2 & 3), precluding calculation and inclusion of YPLL and VSL in the analyses. Another important limitation is the lack of data stratified by both age and sex. As a result, the authors used an average of the male and female period life expectancy when determining YPLL.

**Table 2.** Total years of potential life lost and value of statistical life by age group for Los Angeles County, excluding Long Beach and Pasadena.

Age group	n of deaths	YPLL	VSL (USD)
<5 years old	1	77	$18,520,018
12–17	4	256	$61,694,886
18–29	151	8372	$2,014,986,404
30–49	1532	62,069	$14,938,513,830
50–64	4839	123,370	$29,692,271,530
65–79	8002	112,428	$27,058,745,400
80+	8293	36,614	$8,812,013,590
Missing/unknown	558	–	–
TOTAL	23,380	343,186	$82,596,745,658

USD: US dollars; VSL: Value of statistical life; YPLL: Years of potential life lost.

**Table 3.** Total years of potential life lost and value of statistical life by age group for California State.

Age group	n of deaths	YPLL	VSL (USD)
<5	7	539	$129,640,127
5–17	21	1429	$343,887,496
18–34	918	49,173	$11,834,681,520
35–49	3459	133,760	$32,192,708,640
50–59	6848	185,957	$44,755,492,830
60–64	5886	126,490	$30,443,140,930
65–69	6795	120,034	$28,889,224,760
70–74	7442	104,560	$25,165,106,630
75–79	7606	81,803	$19,687,905,710
80+	24,901	109,938	$26,459,417,630
Missing/unknown	8	–	–
TOTAL	63,891	913,682	$219,901,206,273

USD: US dollars; VSL: Value of statistical life; YPLL: Years of potential life lost.

**Table 4.** Total years of potential life lost and value of statistical life by race and ethnicity among different age groups for California State.

Race/ethnicity	Ages 0–17			Ages 18–34			Ages 35–49			Ages 50–64			Ages 65–79			Ages 80+		
	n of deaths	YPLL	VSL (USD)	n of deaths	YPLL	VSL (USD)	n of deaths	YPLL	VSL (USD)	n of deaths	YPLL	VSL (USD)	n of deaths	YPLL	VSL (USD)	n of deaths	YPLL	VSL (USD)
Latino	14	980	$235,963,564	624	33,425	$8,044,489,403	2473	95,631	$23,016,064,900	8053	205,311	$49,413,486,790	10,515	147,736	$35,556,449,370	7521	33,205	$7,991,698,325
White	5	350	$84,272,701	90	4822	$1,160,262,895	351	13,573	$3,266,736,263	2183	55,656	$13,394,963,580	6269	88,079	$21,198,609,710	10,894	48,097	$11,575,795,980
Asian	3	210	$50,563,621	54	2893	$696,157,737	210	8121	$1,954,457,593	982	25,036	$6,025,585,997	2415	33,931	$8,166,317,187	3880	17,130	$4,122,828,015
African American	3	210	$50,563,621	79	4232	$1,018,452,985	218	8430	$2,028,913,121	863	22,002	$5,295,397,877	1488	20,906	$5,031,668,726	1411	6230	$1,499,306,786
Multi-race	1	70	$16,854,540	10	536	$128,918,099	65	2514	$604,951,160	163	4156	$1,000,173,643	361	5072	$1,220,720,706	374	1651	$397,406,618
American Indian	0	0	0	5	268	$64,459,050	13	503	$120,990,232	56	1428	$343,617,939	84	1180	$284,045,815	73	322	$77,568,671
Native Hawaiian and other Pacific Islander	1	70	$16,854,540	13	696	$167,593,529	32	1237	$297,822,109	104	2651	$638,147,601	130	1827	$439,594,714	67	296	$71,193,164
Other	1	70	$16,854,540	22	1178	$283,619,819	48	1856	$446,733,164	159	4054	$975,629,505	300	4215	$1,014,449,340	308	1360	$327,276,038
Missing/unknown	0			21			49			171			281			373		
TOTAL	28	1960	$471,927,127	918	48,050	$11,563,953,517	3459	131,865	$31,736,668,542	12,734	320,294	$77,087,002,932	21,843	302,946	$72,911,855,568	24,901	108,291	$26,063,073,597

USD: US dollars; VSL: Value of statistical life; YPLL: Years of potential life lost.

VSLY also has important limitations. The VSLY utilized in this paper assumed an age- and income-adjusted but otherwise standard VSL, regardless of factors such as community preferences, values and work-related risk exposures [14,31]. Although the present approach is consistent with prior research [25], and no universally accepted mathematical model of VSLY exists, alternate models of VSLY may incorporate additional variables and arrive at different valuations [31,32] Consequently, the results of this study should be understood as estimates of the financial value of COVID-19 risk reductions across groups, with the trends toward racial and ethnic disparities observed here likely to be more meaningful than precise values. Furthermore, while it is possible that other methodologies would yield less-pronounced racial and ethnic disparities, the current findings may, in fact, be more likely to underestimate the disparities in VSL across racial and ethnic groups, due to the higher COVID-19 exposure risks associated with many low-income jobs, which are often disproportionately held by non-White workers [3]. Finally, it has been argued that VSLY-based cost–benefit analyses should be used with caution in analyses of social distancing measures for COVID-19 [14]. However, a superior metric has not been proposed [14]. Because VSLY remains a broadly understood and accepted tool for evaluating the costs and cost–benefit of risk-reduction interventions [31], the authors feel it is an appropriate and useful strategy for evaluating the financial costs associated with COVID-related deaths.

**Figure 3.**
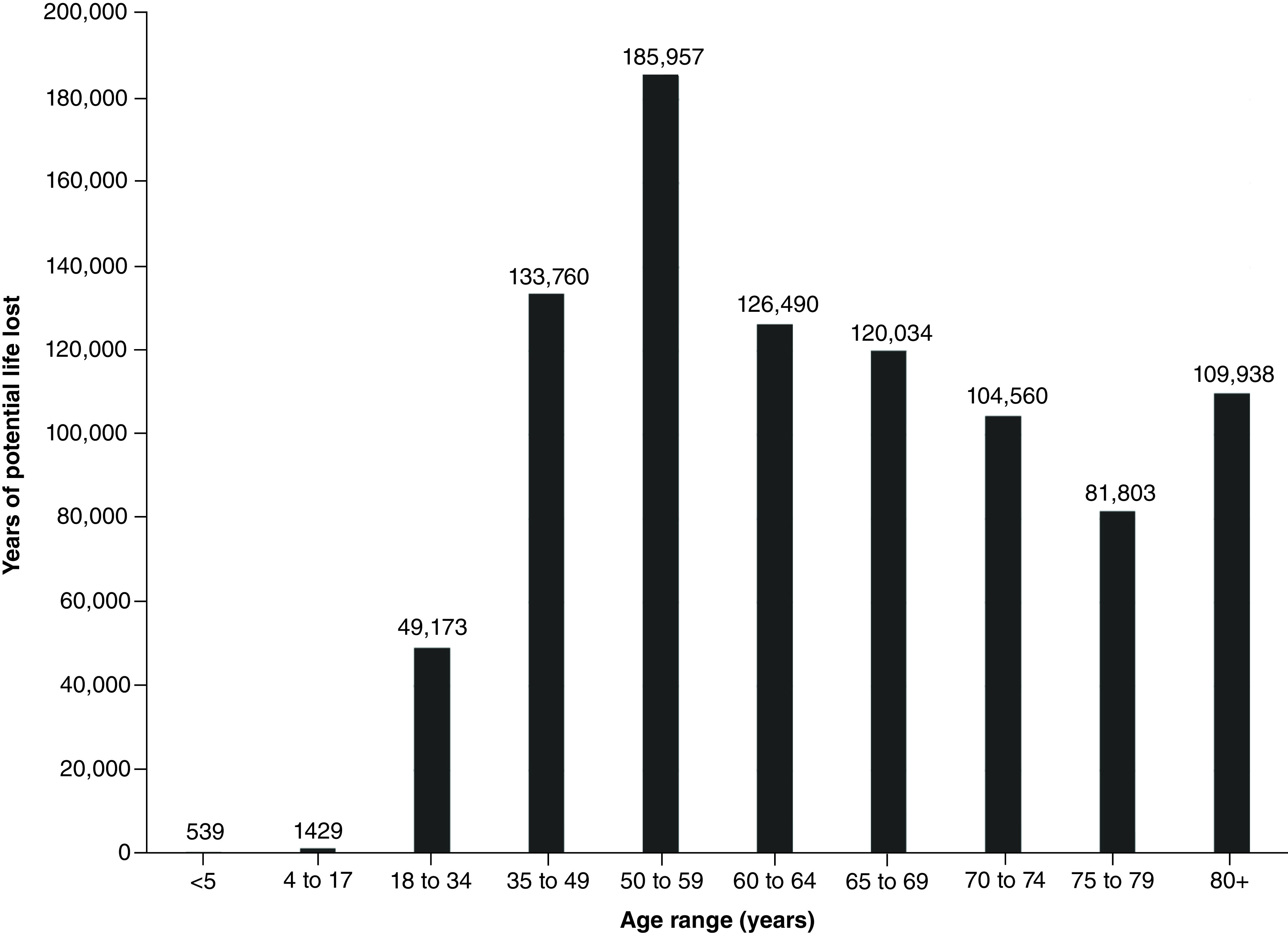
Years of potential life lost by age range for California State.

**Figure 4.**
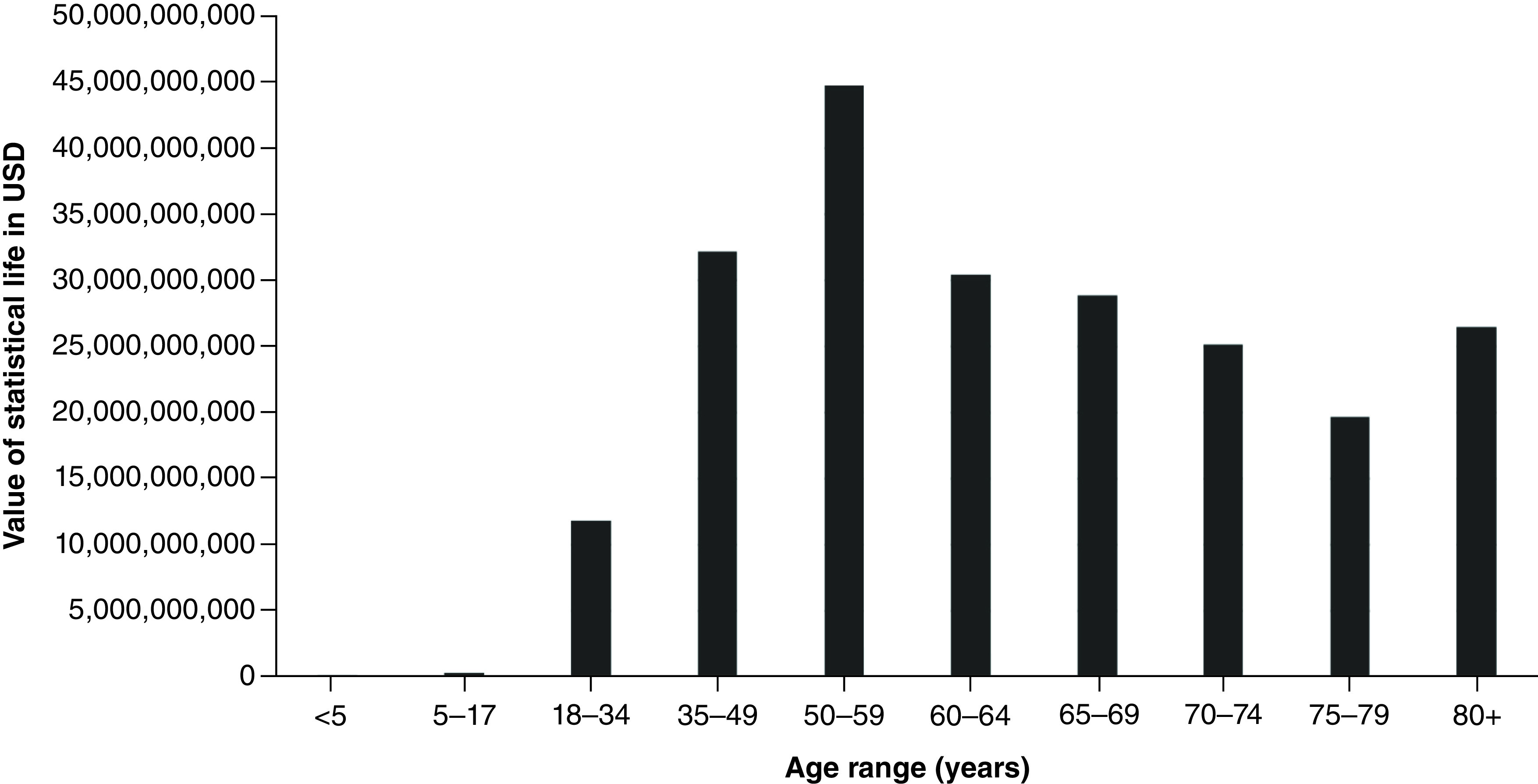
Value of statistical life by age range for California State. USD: US dollars; VSL: Value of statistical life.

**Figure 5.**
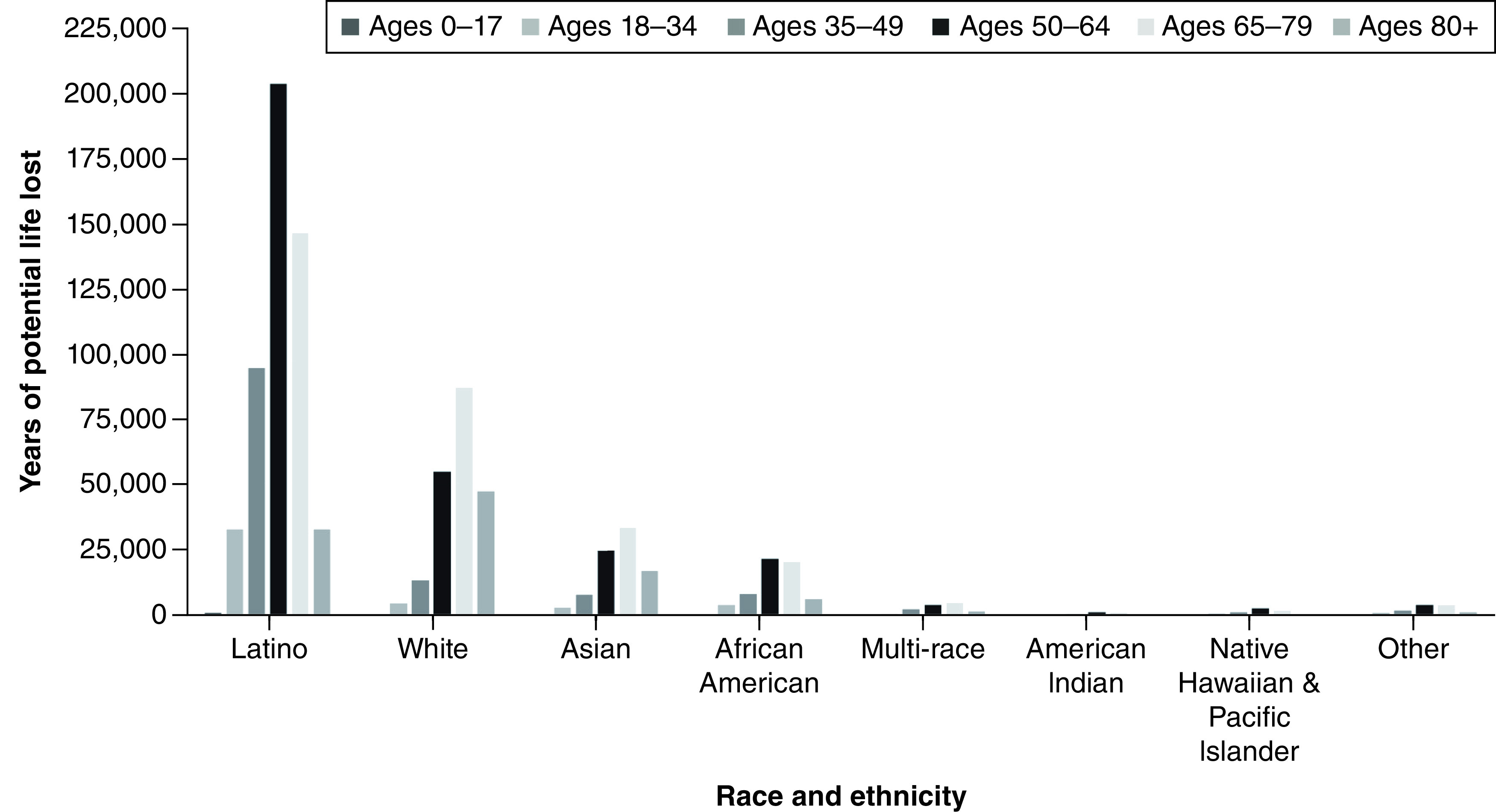
Years of potential life lost by race and ethnicity among different age ranges for California State.

**Figure 6.**
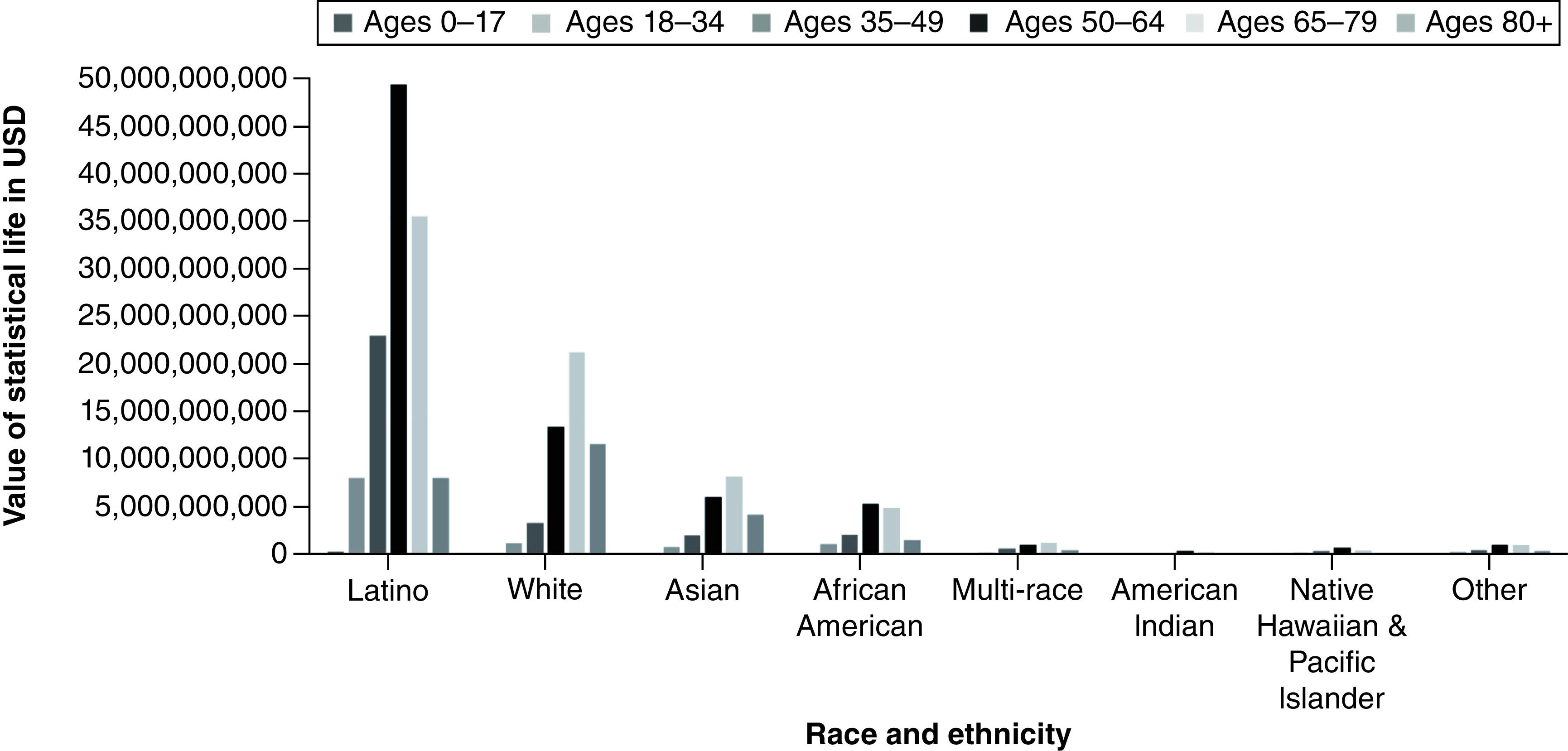
Value of statistical life by race and ethnicity among different age ranges for California State.

## Conclusion

The economic burden of lives lost due to the coronavirus pandemic across California and LA County, excluding Long Beach and Pasadena, is substantial. Among the various age groups, ages 50–64 bore the greatest burden of statistical value of lives lost and estimated YPLL. By race and ethnicity, Latinos experienced the greatest economic burden of lives lost. This study may help inform important policy decisions and community interventions to promote health equity amid an ongoing COVID-19 pandemic, minimize further loss of life and help plan for a post-pandemic future in California State.

**Summary points**

- Estimated years of potential life lost (YPLL) and value of statistical life (VSL) are two measures used to evaluate the impact of excess mortality due to COVID-19.
- The estimated YPLL and the VSL across California and Los Angeles (LA) County due to the coronavirus pandemic are substantial.
- Across California, the economic burden of lives lost has exceeded US$220 billion, with LA County accounting for US$83 billion, as of July 2021.
- The total YPLL across California and LA County was an estimated 913,682 and 343,186, respectively.
- Those aged 50–64 bore the greatest burden of the VSL and the estimated YPLL.
- Latinos accounted for the greatest economic burden of lives lost, with a total YPLL of 516,288 and a total VSL of US$124 billion.
- African Americans were disproportionately impacted by COVID-19, with a higher total YPLL than Asians and Whites.
- These findings may inform important policy decisions to promote health equity amid an ongoing COVID-19 pandemic.
